# Simultaneous procedure transcatheter mitral valve repair and leadless pacemaker implantation under transesophageal echocardiography

**DOI:** 10.1002/joa3.12917

**Published:** 2023-08-27

**Authors:** Ryuki Chatani, Hiroshi Tasaka, Sachiyo Ono, Takeshi Maruo, Kazushige Kadota

**Affiliations:** ^1^ Department of Cardiovascular Medicine Kurashiki Central Hospital Kurashiki Japan

**Keywords:** leadless pacemaker, transcatheter mitral valve repair, transesophageal echocardiography

## Abstract

When we implant leadless pacemaker in patients with contrast agent allergy or poor renal function, the use of sufficient contrast agent is hesitant. Aided by imaging assessment (e.g., intracardiac echocardiography), the procedure may be feasible with a small amount of contrast medium or no contrast medium. In this case, leadless pacemaker implantation was performed at the same time as the transcatheter mitral valve repair, and leadless pacemaker implantation was successful with the use of a very small amount of contrast medium under transesophageal echocardiography guidance.

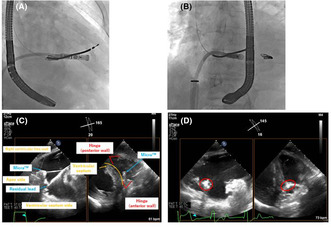

With regard to the leadless pacemaker (Micra™) implantation (LPI) procedure, it is important to avoid cardiac tamponade. It is desirable to confirm that the Micra™ (Medtronic) tip faces the ventricular septum by using sufficient contrast medium in addition to the fluoroscopic image of right anterior oblique (RAO) and left anterior oblique (LAO) view. However, in patients with poor renal function or contrast agent allergy, placement using a sufficient amount of contrast agent is difficult, and placement using an intracardiac echo guide has been reported.^1^ In this case, a patient with mitral regurgitation (MR) and sick sinus syndrome (SSS) with severe renal impairment underwent transcatheter mitral valve repair (TMVr) and LPI under transesophageal echocardiography (TEE) guidance.

An 89‐year‐old man with a history of implantable cardioverter‐defibrillator (ICD) removal due to generator infection was hospitalized for heart failure due to severe MR and SSS type I (Figure 1). His first ICD implanted in the left subclavian pocket removed due to generator infection 11 years ago, and his second ICD implanted in the right subclavian pocket removed due to generator infection 10 years ago. Because of the high surgical risk with a Society of Thoracic Surgeons score of 14.6% and the high risk of generator infection, TMVr with MitraClip™ (Abbott Vascular) and LPI with Micra™ were planned. Because he had poor renal function (creatinine level 3.61 mg/dL, estimated glomerular filtration rate 13.1 mL/min/1.73 m^2^) and LPI under TEE guidance had been reported,^2^ TMVr and LPI were performed at the same time to reduce the amount of contrast medium. Micra™ and MitraClip™ were known to have a very low risk of bloodstream infection,^3^ with residual lead but more than 10 years since the last infection and no recurrence of pocket infection and bloodstream infection. Therefore, we performed the procedure this time.

**FIGURE 1:**
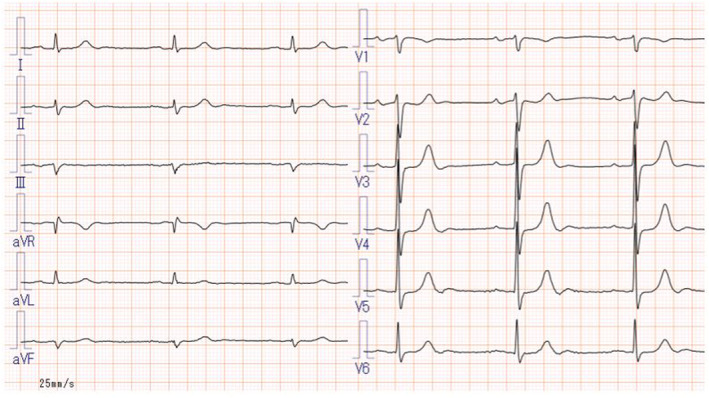
Electrocardiogram findings (HR: 54/min, P‐R interval: 233 ms).

TMVr was performed first because the caliber of the MitraClip™ steerable guide catheter sheath (24 Fr) is smaller than that of the Micra™ introducer sheath (27 Fr) and higher‐intensity anticoagulation is needed for the procedure in the left atrium procedure (target activated clotting time: 250 s for MitraClip™ and 200 s for Micra™). A MitraClip™ was implanted in the middle of A2‐P2 portion and successfully reduced MR to a mild degree. The MitraClip™ steerable guide catheter sheath was replaced with a Micra™ introducer sheath. Deployment was performed three times without contrast medium, but the threshold was unacceptable. At the fourth deployment, a Micra™ was successfully placed under TEE guidance with a small amount of contrast medium (7 mL; Figure 2). The distance to the hinge was checked by TEE to ensure that Micra™ did not face the free wall of the right ventricle. After the sheath was removed, the procedure was completed with a figure‐of‐eight suture. Micra™ pacing rate was set to 70 bpm with VVI mode. The procedure time was 191 min, and the patient was uneventfully discharged on the third day after the procedure (Figure 3). Transthoracic echocardiography 1 month after the procedures showed no findings suggestive of infective endocarditis, and he has been without recurrence of infection. Micra™ ventricular pacing rate was 87%.

**FIGURE 2:**
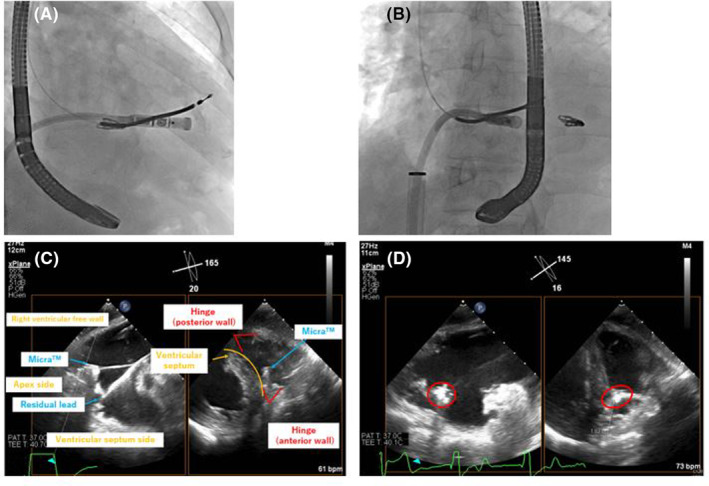
(A) Fluoroscopic image of LPI in RAO view. (B) Fluoroscopic image of LPI in LAO view. (C) TEE image of LPI in transgastric right ventricular inflow view. (D) TEE image of the LP after release in transgastric right ventricular inflow view. The distance between Micra and anterior wall hinge was 18.3 mm. The threshold was 0.75 V at 0.24 ms, R wave 3.5 mV, and impedance 500 Ω.

**FIGURE 3:**
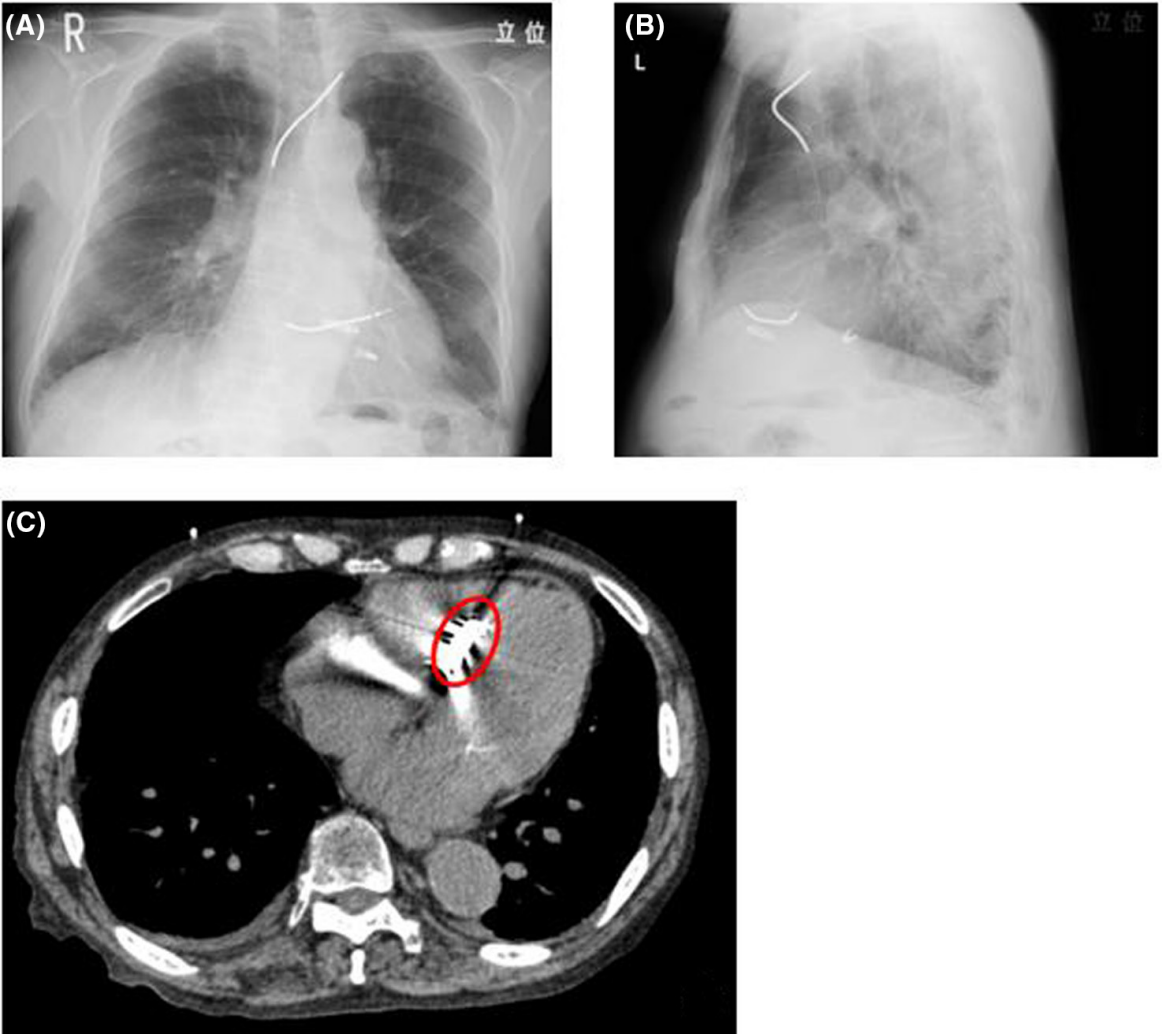
(A) Postoperative chest x‐ray image in frontal view. (B) Postoperative chest x‐ray image in side view. (C) Computerized tomography image of the LP 2 months after the procedure.

There have been case reports of simultaneous percutaneous left atrial appendage closure and LPI,^4^ but to our knowledge, there have been no reports of simultaneous TMVr and LPI. As previously reported, intracardiac echocardiography guidance is also being considered in order to reduce the amount of contrast medium.^1^ However, it requires an additional 8‐Fr access sheath puncture and is not necessarily noninvasive. We could not successfully place without contrast media, using only TEE guidance. Due to residual lead artifacts, it was difficult to evaluate accurately the position of the LP tip. If there are no problems such as residual leads, it may be possible to perform the procedure without contrast medium. Simultaneous TMVr and LPI was a feasible and less invasive approach for patients with severe MR and bradycardia, reducing hospital stay, procedure time, and postoperative rest time.

## CONFLICT OF INTEREST STATEMENT

The authors have nothing to disclose.

## ETHICS APPROVAL STATEMENT

Approval was obtained from the local ethics committee.

## PATIENT CONSENT STATEMENT

Written informed consent was obtained from the patient's relatives for publication of this case report because the patient is deceased.

## CLINICAL TRIAL REGISTRATION

N/A.
